# *Helicobacter pylori *CagA induced interleukin-8 secretion in gastric epithelial cells

**Published:** 2016-12

**Authors:** Zeinab Fazeli, Masoud Alebouyeh, Mostafa Rezaei Tavirani, Masoumeh Azimirad, Abbas Yadegar

**Affiliations:** 1 Faculty of Paramedical Sciences, Department of Basic Sciences, Shahid Beheshti University of Medical Sciences, Tehran, Iran; 2 Foodborne and Waterborne Diseases Research Center, Research Institute for Gastroenterology and Liver Diseases, Shahid Beheshti University of Medical Sciences, Tehran, Iran; 3 *Gastroenterology and Liver Diseases Research Center, Research Institute for Gastroenterology and Liver Diseases, Shahid Beheshti University of Medical Sciences, Tehran, Iran*; 4 *Proteomics Research Center, Shahid Beheshti University of Medical Sciences, Tehran, Iran*

**Keywords:** *Helicobacter pylori*, Cytotoxin-associated gene A, Interleukin-8, CagA variants

## Abstract

**Aim:**

Since, contradictory data have been reported about the effect of diverse variants of *H. pylori *virulence factors on IL-8 induction, we aimed to analyze the effect of this diversity on levels of IL-8 secretion in AGS cell line.

**Background:**

*Helicobacter pylori *colonizes the human stomach and induces the activation of inflammatory cytokines, including interleukin (IL)-8, in the gastric mucosa. This induction promotes neutrophil and monocyte recruitment that causes gastric tissue damage.

**Methods:**

To determine whether different strains of *H. pylori *and their CagA variants have possible roles on IL-8 induction, polarized

AGS cell line was infected with CagA+ *H. pylori *strains carrying different EPIYA motifs (ABCCC and ABC) and CagA- strain for 24 hours. Difference in stimulation of IL-8 was measured by ELISA.

**Results:**

IL-8 secretion was elevated in the treated cells with CagA encoding strains compared with the negative one. Furthermore, a noticeably increased level of IL-8 induction was measured by *the CagA*-EPIYA type ABCCC encoding strain in compare to that carried EPIYA type ABC

**Conclusion:**

Results of this study provide new evidence about different effects of *H. pylori *strains and possible roles of their CagA variants on IL-8 induction. It seems that not only carriage of *cagA *and its expression, but also diversity in EPIYA motif be involved in IL-8 induction in the gastric epithelial cells.

## Introduction

*Helicobacter pylori *is colonized in the stomach of 35%- 70% people worldwide. It is a Gram-negative bacterium that lead to disorders ranging from gastric inflammation to gastric adenocarcinoma (1-4). Several virulence factors of *H. pylori *are involved in the pathogenesis of the stomach.

*H. pylori *chronic infection could increase expression of specific immune mediators, such as interleukins, tumor necrosis factor (alpha), and interferons in the host cells (5-8). Interleukin 8 (IL-8), a potent neutrophil chemotactic factor, plays a crucial role in the proliferation and migration of cancer cells in the infected patients. It also is able to stimulate the host specific inflammatory response through chemo attraction of neutrophils to the site of infected gastric mucosa and their activation (9, 10).

*In vivo *and *in vitro *studies showed higher IL-8 secretion in the *H. pylori *infected cells and mainly correlated this elevated IL-8 level with expression of functional CagA, a major virulence factor of *H. pylori *which is located at the end of the cag pathogenicity island (*cag*-PAI) (11- 16). According to the presence or absence of *cagA*, *H. pylori *strains are classified into two major sub-types, *cagA*-positive or *cagA*- negative (16, 17). Interaction between specific receptors on the gastric epithelial cells and the “needle”-like structure of type IV secretion system, T4SS, CagA is transferred into the cytosol, where it is phosphorylated by host cellular kinases on the repeat sequences known as EPIYA motifs, located in the carboxyl terminus of the protein (18-20). These process lead to an activation of signaling cascades inducing pro- inflammatory cytokines like IL-8(13, 21-23). EPIYA motifs have been classified into four types, based on the adjacent amino acid sequences. The A and B motifs are found in all strains, while the C and D motifsare present within ‘western’ strains and East Asia, respectively(24, 25). It has been suggested that East Asian CagA with one D motif is highly active and it has no requirement to increase its virulence, whereas the less active western type needs to increase its number of tyrosine phosphorylation motifs for being more virulent (26). Besides, the level of CagA phosphorylation depends on the number of EPIYA-C motifs (25, 27-30). It has been shown that co-culture of AGS cell line with Asian strains increases IL-8 secretion on AGS cell line and it may induce more inflammation within the stomach which lead to an increase in atrophic gastritis and gastric cancer (26). Since, there are contradictory reports about the effect of *H. pylori *infection and the number of EPIYA motifs on interleukin (IL)-8 secretion, we aimed to determine the effect of CagA negative and CagA encoding *H. pylori *strains and its variants on IL-8 induction in an in vitro cell culture model.

## Patients and methods

**Strains Used**

*H. pylori *strains HC-113 (complete *cag*-PAI CagA+-EPIYA type ABC, ) and OC-149 (complete *cag*-PAI, cCagA+- EPIYA type ABCCC), as *cagA *positive *H. pylori *strains, and OC-236 (*cag*-PAI- , CagA-), as a *cagA *negative strain, from the microbial collection of Foodborne and Waterborne Diseases Research center, Shahid Beheshti University of Medical Sciences were used for all the analyses(accession numbers JX428768, JX428784).(31)

The strains were recovered from the stocks on Brucella agar medium supplemented with fetal calf serum (10%) (v/v), horse blood (7%), selective supplement (vancomycin 2.0 mg, polymyxin B 0.05 mg and trimethoprim 1.0 mg, Merck, Germany), and amphotericin B (3 mg/l). The plates were incubated at 37 oC for 3 to 5 days in a microaerobic atmosphere (5% O , 10% CO , and 85% N ). The grown organisms were identified as *H. pylori *by Gram staining, colony morphology as well as positive oxidase, catalase and urease reactions.

**AGS gastric epithelial cell co-culture and IL-8 ELISA:.**

The human gastric cancer AGS (ATCC CRL-1739TM) cell line (IBRC, Tehran, Iran) were seeded into six-well plates at a density of 3×105 cells per well and incubated in Dulbecco’s modified Eagle’s medium (DMEM) (Gibco, Grand Island, NY, USA) supplemented with 10% heat-inactivated fetal bovine serum (Gibco, Grand Island, NY, USA), 1% non- essential amino acid (Gibco, Grand Island, NY, USA), 100 U ml−1 of penicillin and 100 *μ*g ml−1 of streptomycin (Gibco, Grand Island, NY, USA) at 37◦C in a humidified incubator (Memmert, Dusseldorf, Germany) containing 5% CO for 2 days before the addition of *H. pylori *strains. For comparison of the effects of CagA positives and negative *H. pylori *strains, gastric epithelial cell lines AGS were infected with a CagA negative *H. pylori *strain and C*agA *positives strains at MOI 100. Briefly, two hours prior to infection, cells were washed with phosphate buffered saline (PBS, 1x) and the medium was replaced with fresh, antibiotic free DMEM media. Bacterial suspensions (100 *μl*) were used to infect gastric epithelial cells in 2 ml total volume. After 24 h incubation, the medium was removed, centrifuged at 15000 g for 10 min and the amount of secreted IL-8 into the medium was determined using Human IL-8 Elisa Ready-set- Go! ® Kit (2nd Generation, eBioscience, Korea), following the manufacturer’s protocols. All samples were measured in duplicate in at least two independent experiments.

## Results

IL-8 levels in AGS cell line

**Figure 1:**
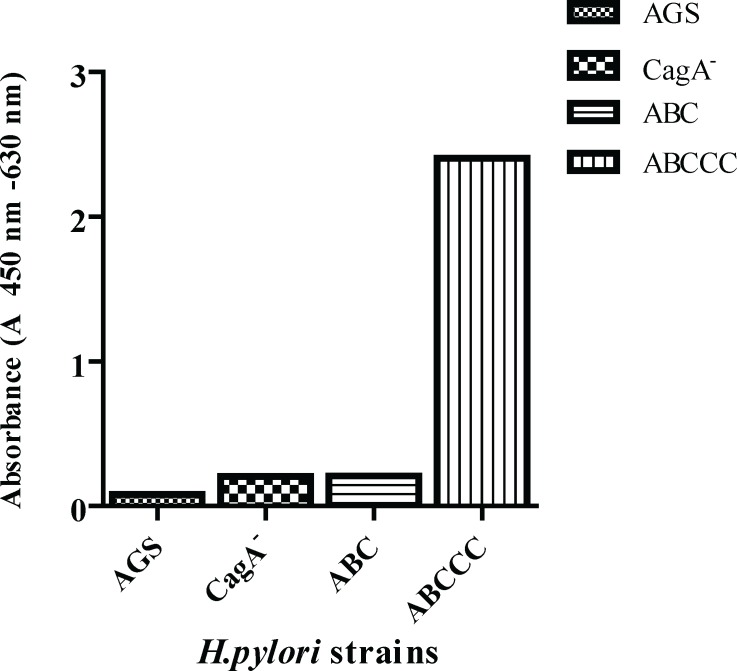
Effect of *H.pylori* strains containing CagA with variable numbers of functional EPIYA motifs on the secretion of IL-8 levels in the supernatants of AGS cells determined by ELISA

In order to quantify the effect of *H. pylori *CagA variants and also and the number of EPIYAC motifs site on IL-8 induction, we infected AGS cells with different strains of *H. pylori*. The tested strains were divided into two groups, according to the presence or absence of *cagA*, positive strains (complete *cag *PAI, CagA EPIYA type ABC and complete *cag *PAI, CagA EPIYA type ABCCC), and negative strain (*cag *PAI-, *cagA*−). After 24 h co-cultured, IL-8 levels were measured as described above.As a control, IL-8 concentration in the supernatants of uninfected AGS gastric epithelial cell lines was determined in the same experimental layout. As presented in Figure 1, the IL-8 secretion was elevated in *H. pylori *infected cells irrespective of the negative or positive strain for *cagA *gene. Furthermore, IL-8 amount was five folds higher in the infected cell line with *cagA *EPIYA type ABCCC in compare to the infected cells with the *cagA*- EPIYA type ABC encoding strain

## Discussion

Our results showed that IL-8 secretion was higher in infected cells in compare to uninfected cells, irrespective of the presence or absence of *cagA *gene. Infection with *H. pylori *is associated with an increased risk of gastric disease. *H.pylori *strains may induce more inflammation within the stomach and this may lead to an increase in atrophic gastritis and gastric cancer. After *H. pylori *infection, a number of genes especially inflammation genes are over expressed in host cells. IL-8, a chemo attractant for neutrophils, is a part of cancer progression. This cytokine release angiogenic growth factors, activates macrophage and immune responses at the tumor site, causes migration and survival of endothelial cells, and potentiates the epithelial-mesenchymal transition (9, 32). Ling, et al. showed that *H. pylori *infection correlates with up-regulated IL-8 expression and its level was found to be associated with invasion, lymph node spreading and clinical stages. These observations indicate that high levels of IL-8 may be associated with a poor prognosis and may be indicative of more aggressive gastric cancer (12). Therefore, it is tempting to consider IL-8 as a prognostic and predictive cancer biomarker. The result of this study also shows that although the amount of IL-8 secreted in this study was not so high, the IL-8 secretion was higher in infected cells with *cagA *positive strains than uninfected or infected cells with a negative one. Since our results demonstrated that CagA*- *EPIYA motifs ABCCC increased IL-8 level in compare to EPIYA motifs ABC, it seems that phosphorylation of CagA at terminal EPIYA-C motifs contributes to IL-8 secretion in AGS cells. However, there are studies that reported contrary results (33-35). Several *in vitro *and *in vivo *studies reported that *H. pylori *induces IL-8 through *cagA*-dependent and -independent manners (30, 36-38). Although different efficiency of IL-8 secretion was observed between different *cagA*-type strains, *cagA *was not always the responsible factor for IL-8 secretion (36,37).

In addition to *cagA*, *H. pylori *peptidoglycans also leading to IL-8 induction through activating NF-kB pathway (13, 18, 39). Papadakos et al showed that following infection of gastric epithelial cells with *H. pylori *strains, a higher activation of NF-kB was observed in the presence on phosphorylated EPIYA-C motifs and in lower levels, in the absence of *cagA *expression and CagA phosphorylation or in the cases where EPIYA-C motifs are totally absent (33) Others proposed that CagA multimerization (CM) motif, the highly conserved amino acid sequence FPLKRHDKVDDLSK, is contributed in IL-8 activation (40,41). Recently it has been suggested that CagL through activation of MAPKs and NF- kB induces secretion of interleukin-8 (IL-8) independently of CagA translocation and peptidoglycan (42). Zhang et al showed that the levels of IL-8 induced by *H. pylori *strains is noticeably differed at an early phase, irrespective of *cagA*- specific sequences. They suggested that other *H. pylori* factors, in addition to *cagA*, affect the enhancement of IL-8 secretion in early *H. pylori *infection phase (24). However, diversity of the level of secreted IL-8 by *H. pylori *remains a controversial issue, as a result of contradictory reports (43- 45). More studies is needed in order to prove this argument. In conclusion, we have demonstrated that the presence of functional CagA protein plays a central role in IL-8 secretion in epithelial cells and the level of IL-8 secreted is amplified with increasing numbers of CagA*-*EPIYAC motifs.
